# Psychometric properties of the scale for non-adherence to antiretroviral medication (NAME) among HIV-infected patients

**DOI:** 10.1186/s13690-019-0382-9

**Published:** 2019-12-06

**Authors:** Zahra Hosseini, Hassan Eftkhar, Teamur Aghamolaei, Abbas Ebadi, Saharnaz Nedjat, Ladan Abbasian, Minasadat hashemi parast

**Affiliations:** 1 Social Determinants in Health Promotion Research Center, Hormozgan University of Medical Sciences, Bandar Abbas, Iran; 2 Department of Health Education and Promotion, School of Health, Tehran University of Medical Sciences, Tehran, Iran; 3 Behavioral Sciences Research Center (BSRC), Life Style Institute, Faculty of Nursing, Baqiyatallah University of Medical Sciences, Tehran, Iran; 4 Department of Epidemiology and Biostatistics, School of Health, Tehran University of Medical Sciences, Tehran, Iran; 5 Iranian Research Center of HIV/AIDS, Iranian Institute for Reduction of High-Risk Behaviors, Tehran University of Medical Sciences, Tehran, Iran; 6 School of Nursing and Allied Medical Sciences Maragheh Faculty of Medical Sciences, Maragheh, Iran

**Keywords:** Barriers of adherence, Antiretroviral medication, HIV, AIDS, Adherence questionnaire

## Abstract

**Background:**

Adherence to HIV medication is necessary for long-term HIV disease management. The objective of this study was to develop and examine the psychometric properties of a scale for measuring the barriers of antiretroviral medication adherence among Iranian Patients.

**Method:**

This was a sequential, exploratory mixed methods investigation composed of two phases. In the qualitative phase, item pool generation and questionnaire designing was carried out through in-depth individual interviews and group discussions; in the quantitative phase, psychometric properties of the questionnaire were assessed. In both phases, the participants were people who were living with HIV/AIDS and were taking antiretroviral medications.

**Results:**

In the first phase, an item pool containing 181 statements related to barriers of adherence to antiretroviral medication was generated. In the second phase, item reduction was applied, and a questionnaire containing 30 items was developed. The psychometric properties of the questionnaire were assessed, which indicated good validity and reliability of the instrument. In exploratory factor analysis, the items were loaded on six factors that altogether explain for 61.98 of the variance. The Cronbach’s alpha and the intra class correlation coefficient of the questionnaire was 0.91 and 0.80, respectively.

**Conclusion:**

This study provided a reliable and valid instrument to identify the barriers of adherence to antiretroviral medication in patients with HIV/AIDS. Identify these barriers and design of interventions to eliminate or reduce of barriers can be an effective means of enhancing adherence to antiretroviral medication among these patients.

## Background

Considering the main routes of transmission of human immunodeficiency virus (HIV), are sexual contact and drug injecting, HIV/AIDS can be classified as a behavioral disease as it relates to the most personal aspects of life. Hence, it is obvious that silence and secrecy are major characteristics of this disease and seeking medical care is avoided. Due to the nature of disease, including the long incubation period (during which there are no signs of the disease), results in delays in the diagnosis of HIV/AIDS and initiation of preventive measures. During this period, patients may unwittingly spread the virus to others [1]. The most common and known antiretroviral medication regimen is highly active antiretroviral therapy (HAART), that considerably limits the spread of the virus in the body [2]. The objective of antiretroviral therapy is preventing disease progression, enhancing the quality of life, and prolonging life. The key to successfully meeting these objectives is the desire and willingness of people with HIV to adhere to the antiretroviral regimen. Adherence to therapy means the correct use of each of the antiretroviral medication doses, at the proper time and exactly as it is prescribed [3, 4]. Based on recent reports, approximately 60% of Iranian patients with HIV/AIDS who are adhere to therapy with antiretroviral medications [5]. Adherence to antiretroviral medications among people living with HIV/AIDS is affected by many different factors. In most studies, the level of adherence to antiretroviral medications is measured [6–8]. Some studies have dealt with effective factors on adherence to HIV medication [8, 9]. There are different questionnaires for the adherence to therapy such as the Morisky questionnaire and self-reported questionnaire assessing adherence to antiretroviral medication and patient medication adherence questionnaire (PMAQ) [10–12]. Also the adherence to medication questionnaire, validated by Ira Wilson’s, is useful for early identification of people who do not adhere to medication [13]. In Iran, Access to information of AIDS patients is restrict because of the social stigma and the need to keep it a secret. Besides, Patients are reluctant to participate in the study because of fear of disease disclosure. so,few studies have been done in Iran on the treatment of patients with HIV/AIDS. In and does not exist a indigenous tool adapted to Iranian culture for check the barriers of adherence to antiretroviral medication. Thus, the main objective of this study was to develop an instrument for measuring the barriers of adherence to antiretroviral medication.

## Method

The sequential, exploratory mixed methods study was conducted in two phases: first, a qualitative study was carried out for item pool generation and designing the instrument; and second, a quantitative approach was used to evaluate the questionnaire.

### First phase: item pool generation and instrument design

Information from in-depth individual interviews and group discussions were used for item pool generation and designing the instrument.

### Participants

The participants had serologic evidence of HIV/AIDS and were being treated with antiretroviral medications. The entrance criteria of the study included having a first-hand experience regarding the study objective (People with HIV/AIDS who are treated with antiretroviral medication and who experience regular or irregular use of these medication and they can give us complete information), being able to speak Persian, and having enough time for interviews or group discussions. Participants were people who were referring to the AIDS center of Imam Khomeini Hospital affiliated to Tehran University of Medical Sciences for taking the antiretroviral medication. Sampling was through a purposeful method with maximum variation so that participants had different experiences regarding HIV/AIDS. The participants represented different age ranges, education levels, occupations, and included both male and female patients. Patients were selected irrespective of whether they had been hospitalized, experienced drug abuse, or demonstrated a good antiretroviral medication adherence.

We used in-depth semi structured interviews with open-ended answers and group discussions for data collection. Usually, the interviews started with the following two questions: (1) How is your life going on with this virus (HIV)? and (2) What therapeutic measures have you taken until now? The subsequent questions were based on the patients’ answers. During the interview we asked the participants for their experiences of illness and therapeutic measures and the factors affecting their adherence to antiretroviral medication. we also used some questions to deepen the interview, such as: what do you mean? Explain more? I understand this from your conversation is it right? After 32 interviews and two group discussions, data saturation was reached. It is noteworthy that we also interviewed two nurses working in infectious ward and one infectious disease specialist.

### Data analysis

Conventional content analysis was applied. The analysis of the data obtained from each interview was carried out before the next interview and it was carried out based on the semantic load of what the participants uttered. Finally, the semantic units were extracted, and each of them became an item of the questionnaire. For example, one of the patients said: “There were many factors that caused me not to take my medications. I was a child, and the school did not register me saying that if I contacted other children they would be infected as well, and this left me with a very bad feeling,” This semantic unit became an item in the questionnaire, which stated: “bad feeling caused by discrimination in comparison with healthy people in the society.” Another patient said: “It was very difficult for me to know I should take medication all during my life.” This statement became an item in the questionnaire, which stated “lifetime medication use.” Finally, a total of 181 items were extracted. After the research team had investigated the items during several sessions, similar items were merged or eliminated, and the semantic and literature structure of the items were modified. Finally, 61 items constituted the basic version of the questionnaire, and the instrument entered the psychometric evaluation phase.

### Second phase: psychometric evaluation of the barriers of antiretroviral medications adherence scale in HIV/AIDS patients

The basic draft of the questionnaire was designed based on the data gathered through individual interviews,group discussions and themes extracted from related studies, which included 61 items with a five-point Likert scale (ranging from very much with a score of 5 to never with a score of 1).

### Face validity

We applied both qualitative and quantitative methods for identifying the face validity. Ten people who were being treated with HAART assessed each item regarding the difficulty level, appropriateness, possible ambiguity, and complexity. For quantitative face validity and impact score (frequency * importance), the same patients were asked to evaluate the instrument and score the importance of each item based on the five-point Likert scale [14].

### Content validity

Qualitative and quantitative content validity was conducted by ten experts (professors in health education and promotion, infection specialists, and professors of nursing in the field of toolmaking), who evaluated the content coverage, compliance with grammar, use of appropriate phrases, and proper placement of items for each aspect. In the quantitative phase, CVI (Content Validity Index) and CVR (Content Validity Ratio) were calculated. For CVR, Lawshe’s method was applied to check how essential an item is for the questionnaire; the experts investigated each item based on a three-part spectrum as follows: is essential, is helpful but not essential, and not necessary. Based on Lawshe’s table, the items with CVR value of 0.62 or above were accepted [15]. For CVI, the same experts checked the items for relevancy, according to Waltz & Bausell’s recommendation and based on a four-point Likert scale scored the items [16].

### Statistical analysis and sampling (construct validity)

Exploratory factor analysis was applied to determine the underlying constructs of the questionnaire. The sample size was calculated based on the number of items (31 items) in the questionnaire. A total of 264 patients with HIV/AIDS treated with HAART for 6 months were selected. After performing the Kaiser-Mayer-Olkin (KMO) index and Bartlett’s test, a principle component analysis with equimax rotation was applied, and the factor loading equal to or greater than 0.3 was considered acceptable.

### Reliability of the instrument

To check the internal consistency of the questionnaire, the Cronbach’s alpha was calculated [17]. Test - Re-test with using intra class correlation coefficient (ICC) was calculated to assess the external consistency of the questionnaire [18]. All statistical analyses were carried out using SPSS software, version 18.

## Results

All 264 study participants completed the questionnaire. The average age of the participants was 39 years (standard deviation [SD] = 9.3 years). The average period of disease diagnosis was 96.81 months. The minimum diagnosis time was 6 months, and the maximum was 384 months or 32 years (SD = 79.03 months). The average time of starting HAART was 61.96 months, and the minimum of having started antiretroviral therapy was 6 months and the maximum was 336 months or 28 years (Table 1).

**Table 1:** Demographic characteristics of the study sample

	Number	Percentage
Sex		
Female	121	45.8
Male	143	54.2
Marital status		
Single	51	19.3
Married	105	39.8
Spouse died	46	17.4
Divorced	62	23.5
Educational level		
Illiterate	11	4.2
Not having high school diploma	119	45.1
High school diploma	97	36.7
Associate degree	20	7.6
Bachelor (B.A., B.S.)	12	4.5
Master degree (M.A., M.S.)	5	1.9
Drug abuse experience		
Yes	103	39
No	161	61

### Face validity

In qualitative face validity, six items that were reported to be difficult to understand were modified. In quantitative face validity, 11 items that had gained scores below 1.5 were eliminated, and finally, 51 items were preserved for the next steps.

### Content validity

To test the qualitative content validity the wordings of some items were modified according to the experts’ opinions. In the quantitative phase, based on Lawshe’s table, 19 items having scores of less than 0.62 were eliminated and the rest of the items saved. In the evaluation of CVI, based on the Waltz & Bausell’s index, all 31 items gained a score of higher than 0.79. Hence, the questionnaire entered the construct validity phase with 31 items.

### Construct validity

Kaiser-Mayer-Olkin (KMO) index and Bartlett’s test of sphericity indicated that the data was appropriate for the factor analysis KMO index = 0.878, χ2 = 4440.737, *P* = 0.000). Based on the results of the analysis, six factors were extracted. As explained in Table 2, each of the six factors had an eigenvalue > 1, and six factors jointly accounted for 61.98% of the variance observed.

**Table 2:** Eigenvalue and variance’s total percentage identified by six extracted factors

Factor	Eigenvalue			Collection of extracted factor loads			Collection of rotated factor loads		
	Total	Variance percentage	Cumulative percentage	Total	Variance percentage	Cumulative percentage	Total	Variance percentage	Cumulative percentage
Personal barriers	9.8	32.66	32.66	9.80	32.66	32.66	4.06	13.53	13.53
Medical challenges	2.37	7.91	40.58	2.37	7.91	40.58	3.64	12.14	25.67
Quality of services	1.87	6.25	46.83	1.87	6.25	46.83	2.90	9.60	35.34
Financial problems	1.97	5.90	52.81	1.79	5.98	52.81	2.83	9.46	44.80
Perceived support	1.48	4.95	57.77	1.48	4.95	57.77	2.64	8.80	53.61
Disclosure of the disease	1.26	4.21	61.98	1.26	4.21	61.98	2.51	8.37	61.98

Among the 31 items, one item was eliminated because of not being congruent with the rest of items and showing low correlation with the rest of the items. Finally, 30 items of the questionnaire were loaded on the six factors (Table 3).

**Table 3:** Factors, items, and extracted factor loads of the factor analysis with equimax rotation

Factor	Items	Factor load
Personal barriers	1. Not admitting the disease and not being able to cope with it	0.751
	2. Lack of mental preparation for taking medicines	0.693
	3. Not being informed of the fact that through using medicines you will experience a normal lifetime	0.683
	4. Lack of awareness of the proper use of the medicines	0.649
	5. Uncertainty about the effectiveness of the medicines	0.630
	6. Considering life and health as unimportant	0.600
	7. Feeling of hopelessness and depression	0.490
	8. Lack of awareness of the risk of developing medicine resistance	0.389
Medical challenges	9. Getting tired of constantly taking medicines	0.477
	10. Large amount of pills to be taken daily	0.732
	11. Being lazy in taking medicines	0.687
	12. Forgetting to take medication	0.645
	13. Medicine’s side effects	0.430
	14. Interference of the medications with the joy received by abusing alcohol and drugs	0.363
services quality	15. Not having enough and proper access to consultation services	0.730
	16. Not receiving empathy from your physician	0.658
	17. Waiting for a long time to receive medicine and visit a doctor	0.516
	18. Lack of needed medicines in therapeutic centers	0.597
	19. Insufficient guidance of medical staff	0.454
	20. Bad behavior of medical staff	0.365
Financial problems	21. The cost of traveling to therapy centers for medicines	0.837
	22. The cost of medical tests	0.779
Perceived support	23. Not having someone to remind you when you should take the medicines	0.845
	24. Not having family care or supervision in taking medicines	0.835
	25. Lack of family emotional support	0.531
	26. Not having Emotional connection with other patients living with HIV	0.406
Disclosure of the disease	27. Fear of the disclosure of the disease while taking medicines	0.757
	28. Bad feeling caused by discrimination in comparison with healthy people in the society	0.707
	29. Fear of others seeing the medicines	0.647
	30. Not going to take the medicines because your privacy is not preserved	0.606

### Internal and external reliability

The Cronbach’s alpha for the entire questionnaire was 0.91 and for the subscales ranged from 0.7 to 0.88. The test - Re-test for the entire questionnaire was 0.80 and for the subscales ranged from 0.57 to 0.75, that providing support to the stability of the questionnaire.

## Discussion

The purpose of this study was to develop and evaluate an instrument for assessing barriers of adherence to antiretroviral medication in HIV/AIDS people. The final questionnaire was checked for validity and reliability with 30 items in 6 subscales (personal barriers, medical challenges, financial problems, perceived support, quality of services, and disclosure of the disease). The CVI and CVR indicated an acceptable validity, and the Cronbach’s alpha and ICC also showed acceptable values, indicating a desirable reliability of the questionnaire. This questionnaire assesses the barriers of adherence to antiretroviral medication among people living with HIV/AIDS.

The subscale of personal barriers indicates the issues that each person experiences independent of others and with minimum effect on the environment. Barriers classified in this category include a lack of awareness and knowledge regarding the disease, therapy and the importance of the regular use of the medications, and depression. Peoples infected with HIV who are aware that not taking their medications or irregularly taking it, will result in the progression to AIDS, will show higher adherence to antiretroviral medications. In addition, depression and hopelessness are quite common among people living with HIV/AIDS, with nearly all participants reporting that they have experienced depression during their disease course, noting that depression decreased their motive for life and hope for the future. The subscale of services quality indicated the existence of some unprofessional behavior from the medical staff, including bad behavior (Rude behavior with insults and disregard to patients) and insufficient consultation, which created challenging circumstances for the patients regarding the start, continuance, and follow-up of their therapy.

The subscale of medical challenges explains the nature of therapy and its influence on the regular use of medications. The obligation of long-term, lifetime use of the medications causes fatigue and forgetfulness of medications doses. Moreover, the medications have some side effects, and it is difficult for the patients to cope with them. All these problems reduce adherence to antiretroviral medications among people living with HIV/AIDS. The subscale of perceived support deals with the fact that these people are rejected and neglected by both their family and the society and are treated unkindly; hence, they lose their motivation for therapy. The subscale of financial problems reveals such problems as reduction of incomes and poverty among these patients that further complicates the process of continuous therapy. Most of the patients dealing with this disease belong to the low socio-economic class and are drug abusers; hence, therapy is not a priority for them and they cannot afford the therapy costs. The subscale of the disclosure of the disease explains the patients’ worries regarding the disclosure of data about their HIV/AIDS status for their family, medical team, and other members of the society, due to the fear of stigma and discrimination.

As per our knowledge, there is no questionnaire that assesses the barriers of adherence to antiretroviral medications hence, we were unable to establish a comparison with the questionnaire designed for this study. However, some questionnaires assess adherence to the therapy; for example, Morisky’s medication adherence scale. This questionnaire is mainly used for chronic diseases and includes four questions: (1) Do you ever forget to take your medicine? (2) Are you careless at times about taking your medicine? (3) Sometimes if you feel worse when you take the medicine, do you stop taking it? and (4) When you feel better, do you sometimes stop taking your medicine? This questionnaire is designed with two option answers (positive or negative), which evaluates the patients’ adherence to therapy, and the number of questions is limited [10].

Our questionnaire investigates different areas with multiple items and solely concentrates on the adherence of people living with HIV/AIDS to antiretroviral medications. The questions presented in the Morisky questionnaire are compatible with some of the items of our questionnaire that are classified under the subscale of medical challenges, including a modified version of the Morisky questionnaire with eight questions [10]. The Morisky questionnaire has been applied in different studies for evaluating the level of adherence to therapy [19–22]. Self-efficacy questionnaire on the use of antiretroviral medication that valided by Joudith. Self efficacy is an important factor in medication use [23]. Our questionnaire does not have a self efficacy sub-scale but some items in sub-scale of Medical challenges, indicates a lack of self efficacy in the patients who that taking antiretroviral medications.

MARS is another questionnaire used for evaluating the level of adherence to therapy among those suffering from psychotic disorders. This questionnaire is designed and psychometrically evaluated by Thompson and includes ten items. In this questionnaire, the patients are required to answer items regarding their attitude and performance of the way they used their medication within the past week. The items of this questionnaire are similar to some of the items of our questionnaire classified under the subscales of personal barriers and medical challenges [24]. Morisky and Thompson questionnaires are not suitable for investigating adherence to antiretroviral medications among people living with HIV/AIDS because AIDS is not similar to other chronic diseases. In Iran AIDS is not only a health problem but also a social dilemma and the society consider the HIV/AIDS patients as sinners, Which causes social deprivation of patients. That’s why patients try to hide their illness.

Each society is based on certain traditional and cultural beliefs. The role and effect of these factors are different in different societies. In Iran, traditional and socio-cultural factors have been integrated and AIDS and its therapy are strongly influenced by mentioned factors. The strength of this study is that the questionnaire is designed based on these factors.

### Limitations

The researchers of this study did their best to design a questionnaire that covers all noted aspects; however, this study also faced some limitations: Talking about AIDS is constrained by some ethical and conventional considerations in the context of Iran. The fact that someone is infected with this virus causes moral stigma in the society, and therefore, it is likely that participants do not express the whole reality about themselves, their disease, and their therapy process. These issues can negatively affect the comprehensiveness of the questionnaire. Moreover, performing additional tests such as confirmatory factor analysis is recommended.

## Conclusion

We believe that this questionnaire provides useful information for the health education and promotion specialists and infectious disease specialists so that they can obtain a deeper and more accurate understanding of the barriers of the adherence to antiretroviral medication among patients with HIV/AIDS and take effective steps for promoting the behavior of adherence to antiretroviral medication.

## Data Availability

The datasets used and analysed during the current study are available from the corresponding author on reasonable request.

## Abbreviations

AIDS: Aquired Immune Deficiency Syndrom
CVI: Content Validity Index
CVR: Content Validity Ratio
HAART: Highly Active Antiretroviral Therapy
HIV: Human Immune Deficiency Virus
KMO: Kaiser-Meyer-Olkin
MARS: Medication Adherence Rating Scale
SD: Standard Deviation
