# Correction to: Psychometric properties of the scale for non-adherence to antiretroviral medication (NAME) among HIV-infected patients

**DOI:** 10.1186/s13690-019-0386-5

**Published:** 2019-12-30

**Authors:** Zahra Hosseini, Hassan Eftkhar, Teamur Aghamolaei, Abbas Ebadi, Saharnaz Nedjat, Ladan Abbasian, Mina Hashemiparast

**Affiliations:** 1 Social Determinants in Health Promotion Research Center, Hormozgan Health Institute, Hormozgan University of Medical Sciences, Bandar Abbas, Iran; 2 Department of Health Education and Promotion, School of Health, Tehran University of Medical Sciences, Tehran, Iran; 3 Behavioral Sciences Research Center (BSRC), Life Style Institute, Faculty of Nursing, Baqiyatallah University of Medical Sciences, Tehran, Iran; 4 Department of Epidemiology and Biostatistics, School of Health, Tehran University of Medical Sciences, Tehran, Iran; 5 Iranian Research Center of HIV/AIDS, Iranian Institute for Reduction of High-Risk Behaviors, Tehran University of Medical Sciences, Tehran, Iran; 6 Department of Public Health, Maragheh University of Medical Sciences, Maragheh, Iran

**Correction to: Arch Public Health (2019) 77:54**

**https://doi.org/10.1186/s13690-019-0382-9**

In the original publication of this article [1] the author pointed out the name of author Minasadat should be ‘Mina Hashemiparast’, not ‘Minasadat hashemi parast’.

The original publication has been corrected.
